# A dielectric and spectrophotometric study of the tautomerization of 2-hydroxypyridine and 2-mercaptopyridine in water†

**DOI:** 10.1039/c9ra08392h

**Published:** 2020-01-13

**Authors:** Biswadeep Bomzon, Yashita Khunger, Ranga Subramanian

**Affiliations:** Department of Chemistry, Indian Institute of Technology Patna 801106 India ranga@iitp.ac.in

## Abstract

The basic ionization (p*k*_1_) and acidic ionization (p*k*_2_) constants and equilibrium constant (*K*_T_) of 2HPy and 2MPy were determined. The p*k*_1_(s) of their *N*- and *X*-methyl derivatives (X = O, S) were also determined. The equilibrium constant of 2MPy is approximately 60 times greater than its oxygen analog, 2HPy. The micro-ionization constants of the functional groups, –NH (p*k*_A_ and p*k*_C_) and –XH (p*k*_B_ and p*k*_D_), were determined to provide further insights into the ionization equilibria of these N-heteroaromatic XH compounds (2HPy and 2MPy). The relaxation time of water (*τ*) in aqueous solutions of 2HPy and 2MPy are collectively used with the *K*_T_ values to determine the forward (*k*_f_) and backward (*k*_b_) rate constants of tautomerization. Subsequently, the *k*_f_ and *k*_b_ are used to provide the rationale for the *K*_T_ and *τ* values.

## Abstract 2

The basic ionization (p*k*_1_) and acidic ionization (p*k*_2_) constants and equilibrium constant (*K*_T_) of 2HPy and 2MPy were determined.

## Graphical abstract

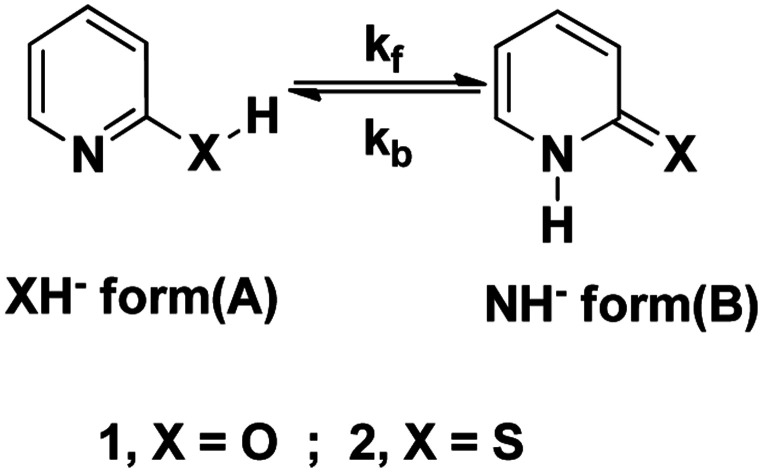

## Introduction

Tautomerism is the dynamic equilibrium between molecules with the same atomic composition and the proton transfer between two electronegative atoms within the same molecule. The interconversion is associated with a small potential energy barrier.^1,2^ Research on tautomerism covers scientific fields ranging from chemistry, biochemistry to chemical physics. These studies are of fundamental importance in the interpretation of reaction mechanisms involving tautomers, such as to stabilize covalent organic framework;^3^ in designing computer-aided drug and their biological effects,^4,5^ and as molecular switches.^6,7^

**Fig. 1:**
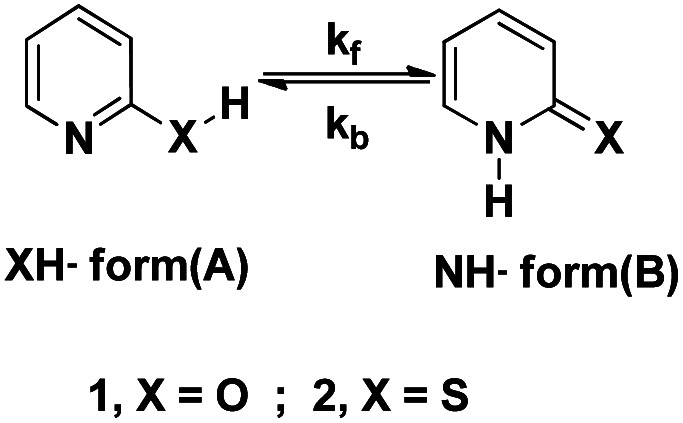
Tautomerization of N-heteroaromatic XH compounds.

The keto–enol tautomerism process in various systems (especially 2-pyridone, 2Py) has received attention as one of the simplest prototype systems to study proton shuttling, hydrogen bonding, and tautomerism mechanisms. Fig. 1 illustrates the rapid and facile tautomeric transformations. Indeed, 2Py is a heteroaromatic system to describe hydrogen-bonded DNA base pairs;^8^ catalytic design element;^9^ with potential for self-assembly platform of bidentate ligands^10^ and blocks in synthesis.^11^ Some of the nucleic acid bases exhibit the keto–enol tautomerism, in which proton transfer takes place between N and O sites of the molecules and may be one of the possible routes for the DNA and RNA mutations.^12–14^ From the theoretical and experimental studies that have been reported,^15–23^ it can be gleaned that in pyridine and pyrimidine substituted at 2- or 4- positions by tautomeric groups XH (X = O or S) exist in lactam or thione forms rather than in lactim or thiol forms.

The rate constants (*k*_f_ and *k*_b_) of tautomerization are critical parameters to determine the time required to attain the state of equilibrium between the tautomers. The rate constants also provide valuable insights on the stabilities of the tautomeric forms and their respective concentrations in the system. The tautomeric concentrations in the system are crucial since it has been found that several models of spontaneous mutations in DNA involve the role of minor tautomeric forms.^14,24^ It has also been reported that the minor tautomeric forms are included in the stabilization of anomalous DNA structures.^25–30^ The rate of mutation tends to increase with the increase in the formation of minor tautomers, thus any situations that would cause the high mutation rates can be avoided for the genetic material.^31^ Analysis of rate constants of tautomerization can also be constructive in the development of lethal antiviral mutagenesis for the treatment of virus-like HIV.^32^

The tautomers present in the solution containing more than one functional group would gain or lose protons, depending on the pH of the medium. This ionization of functional groups occurs at the molecular level, cannot be determined empirically and is called micro-ionization constants. On the contrary, the macro-ionization (ionization) constants can be measured using a potentiometric method, a spectrophotometric method, or by NMR. The ionization constants correspond to the simultaneous ionization of functional groups and are hybrid values of micro-ionization constants.^33^ There is a negligible influence of acid–base equilibria on the stabilization of the physiological structures of DNA since the nucleic acid bases are neutral under normal physiological conditions. Nevertheless, the evaluation of ionization and microionization constants cannot be taken for granted, for it has been suggested that the ionization may be relevant in deciding the mutagenic properties of the analogs of nucleic acid bases.^34^ It is also reported that the DNA polymerase can merge the ionized base pairs into DNA.^35^

Dielectric Relaxation Spectroscopy (DRS) is a tool to study the structure and dynamics of the complex liquid fluids whose properties are dominated by hydrogen bonding and is adequate for an investigation of hydrogen bond rearrangement dynamics. DRS has been widely applied for investigating pure solvents,^36–42^ solvent–solvent mixtures,^43^ super-cooled, and glass-forming liquids,^44–48^ water–organic compound mixtures,^49–60^ and electrolyte solutions. In-depth knowledge in hydrogen bond is required to understand the nature of molecular interactions taking place in biological activities; enzyme catalysis, drug synthesis and chemical and electrical properties of the material.^61–68^

2-Hydroxypyridine (2HPy) (1A ⇌ 1B) and 2-mercaptopyridine (2MPy) (2A ⇌ 2B) in aqueous solutions have been studied, experimentally^69–73^ and theoretically.^74–81^ To the best of our knowledge, this is the first study that determines the rate constants, *k*_f_, and *k*_b_ (of tautomerization) in aqueous medium, as depicted in Fig. 1.

The spectrophotometric approach is employed to determine the acidic and basic ionization constants, *k*_1_ and *k*_2_, of 2HPy and 2MPy along with the basic ionization constants of their respective *N*-methyl and *X*-methyl derivatives, *k*_1(NMe)_ and *k*_1(XMe)_. The equilibrium constant (*K*_T_) of aqueous solutions of 2HPy and 2MPy was determined using ionization constant. The equilibrium constants, in turn, were used to calculate the micro-ionization constants, *k*_A_, *k*_B_, *k*_C_, and *k*_D_, of the NH and XH functional groups present in both 2HPy and 2MPy. The outcomes of the spectrophotometric approach alone were inadequate to provide the values of the *k*_f_ and *k*_b_ directly. DRS is used to investigate the tautomerization of aqueous solutions of 2HPy and 2MPy. DRS provides the relaxation time in an aqueous solution of 2HPy (*τ*_2HPy_) and 2MPy (*τ*_2MPy_), which were used to find the *k*_f_ and *k*_b_ of tautomerization. Thus, DRS, together with spectrophotometry, were combined to determine the rate constants of tautomerization, *k*_f_, and *k*_b_, for the first time.

## Results and discussions

**Fig. 2:**
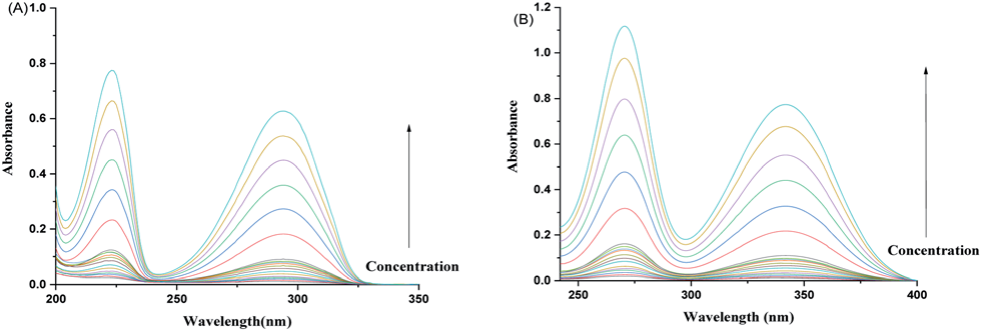
Absorption spectra of aqueous solutions of (A) 2HPy and (B) 2MPy in the concentration range of (0.168–11.776) × 10^−5^ M.

For 2HPy, and 2MPy, aqueous solutions are prepared in the concentration range of 0.168 × 10^−5^ to 11.777 × 10^−5^ M, in such a way that the absorbance values of these solutions remain lower than or closer to unity (see Fig. 2).

**Fig. 3:**
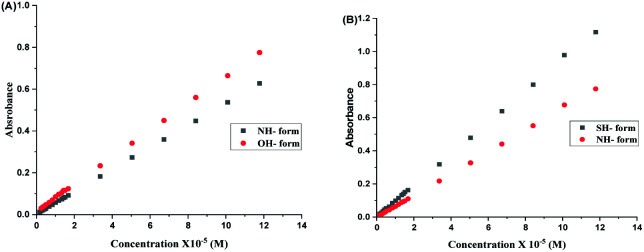
Concentration (× 10^−5^ M) *versus* absorbance plots of aqueous solutions of (A) 2HPy and (B) 2MPy.

Fig. 2 indicates the presence of two *λ*_max,_ which corresponds to the two tautomers, NH- and XH forms. It can also be inferred from the Fig. 2, that the dimers (both homo- and hetero-) are absent in the system. As a consequence, it can be drawn that the tautomers of both 2HPy and 2MPy prefer to form hydrogen bonds (H-bonds) with water molecules rather than to make H-bonds between them. Indeed the self-association of tautomers in water is thermodynamically unfavorable.^88^ Therefore, the tautomerization of N-heteroaromatic XH compounds can be presented as in Fig. 1, without any elaborations. Comparing Fig. 2a and b, *λ*_max_ corresponding to XH and NH forms, shifts towards longer wavelength (lower energy difference) from 2HPy (223 nm; 294 nm) to 2MPy (271 nm; 342 nm). This shift in the *λ*_max_ might be because the polar solvent (H_2_O) stabilizes the electronic ground states of 2HPy, thereby increasing the energy difference between the concerned electronic states of 2HPy in comparison to the 2MPy molecules.^89^ From Fig. 3, it's clear that the systems of our interest follow the Beer–Lambert's law. The experiment result (see, Fig. 2(A) and 3(A)) also reveals that the OH-form of 2HPy is detected only from 0.252 × 10^−5^ M. It's probably the OH-form is undetectable at lower concentrations (below 0.252 × 10^−5^ M) in comparison to the NH-form in aqueous solutions. In the case of 2MPy, both peaks are present at lower concentrations. This result is consistent with the molar absorption coefficient values of 2MPy; *ε*_2MPy_ is higher than the molar absorption coefficient of 2HPy, *ε*_2HPy_ (Fig. 2).^21,90^

**Fig. 4:**
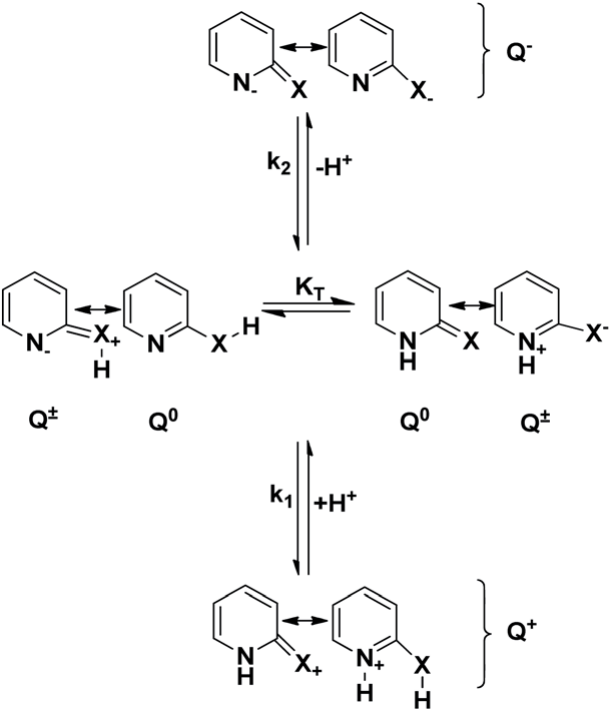
Ionization equilibria of N-heteroaromatic XH compounds. Q^0^ represent the neutral forms, and Q^±^ represent the zwitterionic form.

Spectrophotometric measurements allow us to assess the acidic (*k*_2_) and basic (*k*_1_) ionization constants by addition of protons (H^+^) and hydroxyl ions (OH^−^) to form the cationic (Q^+^) and anionic forms (Q^−^) respectively, of N-heteroaromatic XH compounds respectively (see Fig. 4).

Applying eqn (7), the p*k*_2_(s), of 2HPy, and 2MPY were calculated (see Table S2 and S3†), then using eqn (9), p*k*_1_ of 2HPy was obtained. Likewise, the basic ionization constants of 2-methoxypyridine (p*k*_OMe_) and *N*-methyl-pyridone (p*k*_1(NMe)_) were also obtained using eqn (9), and the basic ionization constant of 2-methylthiopyridine (p*k*_SMe_) was evaluated using eqn (10) as given in Tables S1, S4, S5, and S6† respectively. Table 1 provides the ionization constants, p*k*_1_, p*k*_2_, p*k*_1(NMe)_, and p*k*_1(XMe)_, and the calculated values of *K*_T_(s). The equilibrium constant (*K*_T_) of the tautomerism (A ⇌ B), of N-heteroaromatic XH-compounds in aqueous solutions, are evaluated from the ionization constants of 2HPy and 2MPy and their *X*- and *N*-methyl derivatives using eqn (4) or (5).^91,92^

**Table 1:** The ionization constants (*k*_1_ and *k*_2_), of 2HPy and 2MPy, their *X*- and *N*-methyl derivatives (*k*_1(XMe)_ and *k*_1(NMe)_) and the equilibrium constants (*K*_T_ = [B]/[A]) in the water at room temperature

*k*_1_, *k*_2_, *K*_T_, Δ*G*	2-Hydroxypyridine	2-Mercaptopyridine
p*k*_1_	0.76 ± 0.01 (0.75)	−1.38^a^ (—)
p*k*_2_	12.14 ± 0.02 (11.62)	10.10 ± 0.01 (9.81)
p*k*_1(XMe)_	3.25 ± 0.01 (3.28)	3.37 ± 0.01 (3.59)
p*k*_1(NMe)_	0.31 ± 0.01 (0.32)	—
*K*_T_	912 ± 29 (910)	56 230 ± 1000 (70 000)
log *K*_T_	2.96 ± 0.01 (2.96)	4.75 ± 0.01 (4.82)
Δ*G* (kJ mol^−1^)	−16.88 ± 0.06 (−16.90)	−27.09 ± 0.06 (−27.50)

a From ref. 17. The literature values are listed in the parenthesis.

Table 1 shows that p*k*_1_, p*k*_1(XMe)_, p*k*_1(NMe)_ of 2HPy are in agreement with the literature values while p*k*_2_ of 2HPy and p*k*_2_, p*k*_1(XMe)_ of 2MPy are close to the literature values.^19,83,84,92^*K*_T_ and Δ*G* values of 2HPy and 2MPy are comparable to the literature values.^19,83,93^ The *K*_T_ and Δ*G* values of the 2HPy and 2MPy show that the NH- tautomers are predominant over the XH- tautomers.^94–96^ As a consequence, we can infer that the forward rate constant, *k*_f_, is higher than the backward rate constant, *k*_b_, of N-heteroaromatic XH compounds in water. Further, the *K*_T_ and Δ*G* of 2MPy are approximately 60 and 1.6 times greater than that of 2HPy. This difference is probably due to the differences in the values of their rate constants, *k*_f_, and *k*_b_. The p*k*_1_ and p*k*_2_, determined experimentally, are composite and are related to the micro-ionization constants of the individual functional groups –NH (*k*_A_ and *k*_C_) and -XH (*k*_B_ and *k*_D_) of the N- heteroaromatic compounds by the following expressions.^33,83^

1 *k*_1_= *k*_A_+ *k*_B_

2 1/*k*_2_ = 1/*k*_C_ + 1/*k*_D_

3 *k*_1_*k*_2_ = *k*_A_*k*_C_ = *k*_B_*k*_D_

**Fig. 5:**
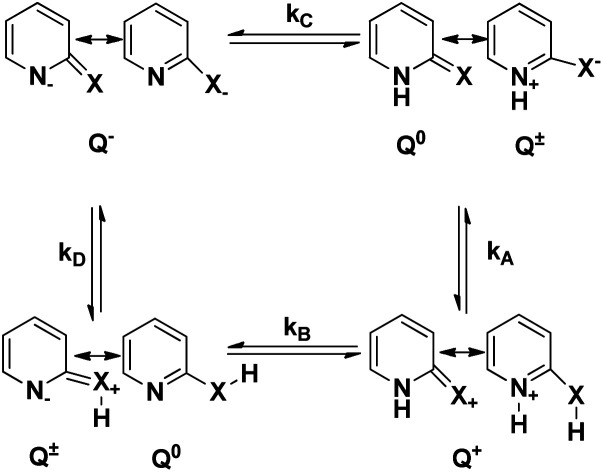
Micro-ionization equilibria of N-heteroaromatic XH compounds. Q^−^ represents the anionic forms, and Q^+^ represents the cationic forms of the tautomers.

The micro-ionization constants are not independent of each other since the ionization of their respective functional groups can follow either of the two paths depicted in Fig. 5.

In the case of 2HPy, the basic micro-ionization constants of the NH and XH tautomers (*k*_A_ and *k*_B_) are assumed to be equal to the basic ionization constants of *N*-methyl (*k*_1(NMe)_) and *X*-methyl (*k*_1(XMe)_) derivatives.^83^Eqn (4) gives the relationship between *K*_T_ and the microionization constants (*k*_C_ and *k*_D_).

4 *K*_T_ = *k*_f_/*k*_b_ = [NH form]/[XH form] = *k*_A_/*k*_B_ = *k*_D_/*k*_C_ = *k*_1(NMe)_/*k*_1(XMe)_

On the other hand, for 2MPy, the *K*_T_ was determined by employing the basic ionization constants of 2MPy (*k*_1_) and its *X*-methyl derivative (*k*_1(XMe)_) by the following equation.^83^

5 *K*_T_ = (*k*_1_/*k*_1(XMe)_) − 1

The micro-ionization constant of the tautomers of both 2HPy and 2MPy are calculated from the values given in Table 1, using eqn (1)–(4) and is shown in Table 2.

**Table 2:** Micro-ionization constants of XH (*k*_B_ and *k*_D_) and the NH- tautomers (*k*_A_ and *k*_C_) of the 2HPy and 2MPy in the water at room temperature^a^

Micro-ionization constants	2-Hydroxypyridine	2-Mercaptopyridine
p*k*_A_	0.76 ± 0.02 (0.75)	−1.38 ± 0.01
p*k*_B_	3.72 ± 0.01 (3.71)	3.37 ± 0.01
p*k*_C_	12.14 ± 0.02 (11.62)	10.10 ± 0.01
p*k*_D_	9.18 ± 0.03 (8.66)	5.35 ± 0.01

a The values of p*k*_A_ and p*k*_B_ for 2HPy are identical to the literature values, and p*k*_C_ and p*k*_D_ are comparable.^82^ The p*k*_A_, p*k*_B_, p*k*_C_, and p*k*_D_ of 2MPy are reported for the first time in this work.

**Fig. 6:**
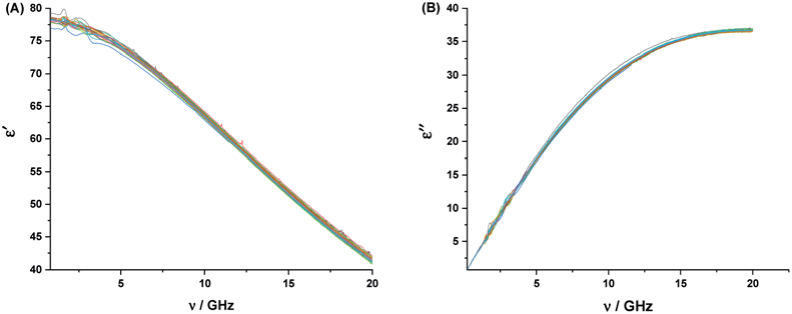
Real (*ε*′) (A), and imaginary (*ε*′′), (B), parts of the complex dielectric function for aqueous solutions of 2HPy at the concentration range of 0.168–1000 × 10^−5^ M, at room temperature.

**Fig. 7:**
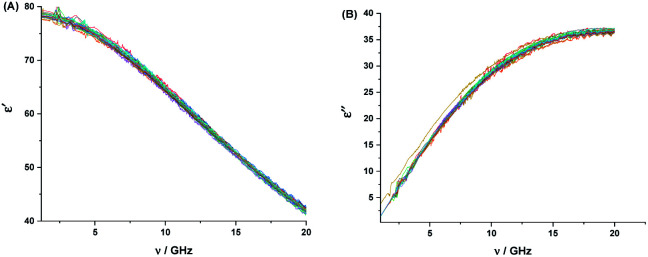
Real (*ε*′) (A), and imaginary (*ε*′′), (B), parts of the complex dielectric function for aqueous solutions of 2MPy at the concentration range of 0.168 × 10^−5^ to 1000 × 10^−5^ M, at room temperature.

The aqueous solutions of 2HPy and 2MPy in the concentration range of (0.168–1000) × 10^−5^ M were used to record the DRS at room temperature. The real (*ε*′) and imaginary (*ε*′′) parts of the dielectric spectra of these solutions were plotted against the frequency (*ν*). Fig. (6) and (7) show the plots, respectively.

**Fig. 8:**
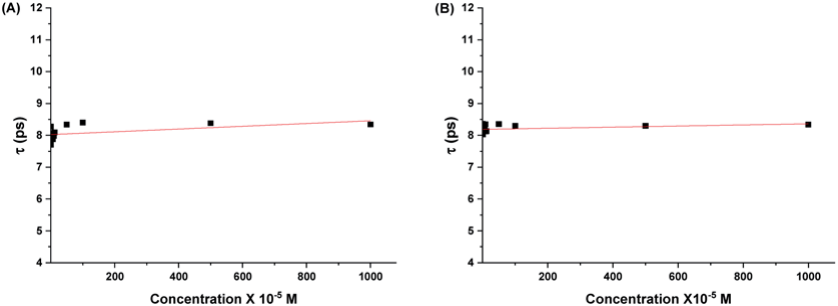
Relaxation times of water, *τ* against the concentration of aqueous solutions of (A) 2HPY, and (B) 2MPy.

From Fig. (6) and (7), it is observed that both the real and imaginary parts of the dielectric spectra of the aqueous solutions of both 2HPy and 2MPy resemble the pure water. We had varied the concentrations from (0.168 to 1000.00) × 10^−5^ M; their spectra are inseparable from pure water as well as from each other. It is inferred that the water domain changes very little with the addition of both 2HPy and 2MPy. Thus, the one-term Debye function can be applied to the dielectric spectra conveniently during fitting. The error to the parameters, *ε*_α_, Δ*ε*, *ε*_s_, and *τ*, were determined using error analysis^97^ and are given in parenthesis in Tables S15 and S16.† Tables S15 and S16† reveal that the *ε*_α_, Δ*ε*, and *ε*_s_ are close to values of pure water. The *τ*_w_ values decrease only slightly from that of pure water. The *τ* remains virtually unchanged with the concentration variations for 2HPy solutions and 2MPy solutions in water. The *τ* values indicate that the *τ* of 2HPy and 2MPy is independent of the concentrations of the solutes, as shown in Fig. 8. As a consequence, the *τ* can be expressed as the reciprocal of the sum of the *k*_f_ and *k*_b_ as shown below,^98^

6 *τ* = 1/(*k*_f_ + *k*_b_)

Fig. 8 shows a linear fit of *τ* and concentration. From the fit, we get intercepts for 2HPy of 8.02(3) ps and 2MPy of 8.19(2) ps with slope nearly zero. Indeed, this implies DRS provides a crucial relation between the *τ*, and *k*_f_ and *k*_b_. By combining eqn (11) and (13), the values of rate constants, *k*_f_, and *k*_b_ are evaluated (see Table 3).

**Table 3:** The relaxation times (*τ*) of water and rate constants, *k*_f_ and *k*_b_, of tautomerization (A ⇌ B) in aqueous solutions of 2HPy and 2MPy

Compounds	*τ* (ps)	*k*_f_ (in s^−1^)	*k*_b_ (in s^−1^)
2HPy	8.02(3)	(1.24 ± 0.05) × 10^11^	(1.36 ± 0.04) × 10^8^
2MPy	8.19(2)	(1.22 ± 0.04) × 10^11^	(2.18 ± 0.04) × 10^6^

From Table (3), it is seen that the *k*_f_ values of tautomerization of both 2HPy (1A ⇌ 1B) and 2MPy (2A ⇌ 2B) are higher than the *k*_b_ values since the NH-forms are more stable than their XH-forms, due to the fact that the free X-atoms are readily available for stronger hydrogen bonding with water molecules.^99^ The similarity of the *k*_f_ values of both the N-heteroaromatic XH compounds can be explained because their stability of NH-forms in water is almost identical. The *k*_b_ values of 2HPy are higher than that of 2MPy by a magnitude of about 60. The difference in the *k*_b_ values can be explained on the basis that the XH forms of 2HPy are more stable than the same form of 2MPy in water. Since the OH group forms stronger hydrogen bonds with water molecules, which increases the stability of XH-form of 2HPy in water and, the SH group, on the other hand, cannot form strong hydrogen bonds with water molecules. So, the XH form of 2MPy is less stable.

## Conclusions

The aqueous solutions of both 2HPy and 2MPy obey the Debye equation. The NH-forms of both 2HPy and 2MPy are predominant over their XH-forms. The NH-tautomer forms stronger hydrogen bonds with water molecules than their XH-tautomers. The equilibrium constant (*K*_T_) of 2MPy (2A ⇌ 2B) is approximately 60 times greater than the equilibrium constant (*K*_T_) of 2HPy (1A ⇌ 1B). The backward rate constant (*k*_b_) of 2HPy is higher than that of 2MPy by the value of 60, while the forward rate constant (*k*_f_) is identical. The NH-forms have equivalent stabilities, while the XH-form of 2HPy is more stable than the same form of its sulfur analog, 2MPy. The XH-form of 2HPy forms stronger hydrogen bonds with water molecules through –OH groups. The –SH groups do not form stronger hydrogen bonds with water molecules; hence, XH-form of 2MPy is less stable.

## Experimental section

Commercially purchased 2HPy (Alfa Aesar, 98+ %), 2MPy (Alfa Aesar, 98%), 2-(methylthio) pyridine (Sigma Aldrich, with ≥ 95%), *N*-methyl-2-pyridone (Sigma Aldrich, with ≥ 99%) and 2-methoxypyridine (Sigma Aldrich, with 98%) was used. The solutions, in molarity (M), were prepared using triple distilled water. The pH(s) of the prepared solutions and buffers were measured using a digital pH meter, PC 2700-Oakton, and a combined glass pH electrode. Commercial standardized buffer solutions of pH = 4, 7, and 10 were used for calibration of the combined electrode. Buffers (0.01 M) of low ultraviolet absorption (glycine, borate, acetate, and formate) were used. UV-Visible spectrophotometer, Shimadzu (UV- 2550), was used in spectrophotometric measurements (200–400 nm). The sample cuvettes used are of quartz (Hellma Analytics) with 1 cm path length.

The aqueous concentrations in the range of 10^−4^ to 10^−5^ M were used for accurate determination of the ionization constants.^82^ The literature values of ionization constants for different compounds, determined from the potentiometric techniques^19,83,84^ were chosen as the estimated values of ionization constants. Appropriate buffers were prepared for the determination of absorbances of the neutral forms. Suitable concentrations of the base (NaOH) and acids (HCl, H_2_SO_4_) were also used in preparing the solutions for the determination of absorbances of ionic forms of the compounds. The different solutions the compounds were probed in the range of 200–400 nm against their suitable blanks with an identical pH. The analytical wavelengths were chosen based on the highest difference between the absorbances of the neutral and ionic forms. The ionization constants (p*k*_1_, p*k*_2_, p*k*_1(NMe)_, p*k*_1(XMe)_) were determined by employing the appropriate eqn (7)–(10).

For acidic ionization constants, p*k*_1_,

7 when *A*_I_ > *A*_M_, p*k*_1_ = pH + log(*A*_I_ − *A*)/(*A* − *A*_M_)

8 when *A*_M_ > *A*_I_, p*k*_1_ = pH + log(*A* − *A*_I_)/(*A*_M_ − *A*)

For basic ionization constants, p*k*_2_,

9 when *A*_I_ > *A*_M_, p*k*_2_ = pH + log(*A* − *A*_M_)/(*A*_I_ − *A*)

10 when *A*_M_ > *A*_I_, p*k*_2_ = pH + log(*A*_M_ − *A*)/(*A* − *A*_I_)

where *A*_I_ is the absorbance of the ionic forms, *A*_M_ is the absorbance of a neutral form, and A is the absorbance of species in the buffer solutions.

Agilent E5071C Vector Network Analyzer (VNA) with an Agilent E85070E dielectric probe kit was calibrated against the calibration standards, air, short circuit, and water. The Agilent 85070E dielectric software was used to measure the complex permittivity spectra of the pure triple distilled water and various aqueous solutions of 2HPy and 2MPy, in the frequency range of 88 MHz to 20 GHz at room temperature.

The real and imaginary dielectric spectra, obtained from the VNA were fitted simultaneously using a non-linear least square method based on the Levenberg–Marquardt algorithm^85^ in Origin 2018b according to the Debye equation, as shown below,

11 ε*(*ν*) = *ε*′ − i*ε*′′ = *ε*_α_ + Δ*ε*/(1 + 2πi*ντ*)

where, i = √−1, *ν* = frequency (in GHz), *ε*_α_ = high frequency dielectric permittivity, Δ*ε* = *ε*_s_ − *ε*_α_ = relaxation amplitude, *ε*_s_ = static permittivity, *τ* = relaxation time of water (in ps). It is familiar that pure water conforms to eqn (11) (ref. 41 and 86), but the aqueous solutions of 2HPy and 2MPy may not necessarily agree with the Debye function. Thus, to verify our concern, *ε*′(*ω*) against *ε*′′(*ω*)/*ω*, and *ε*′(*ω*) against *ωε*′′(*ω*) were plotted according to the eqn (12) and (13), which are rearranged versions of Debye equation.^87^12
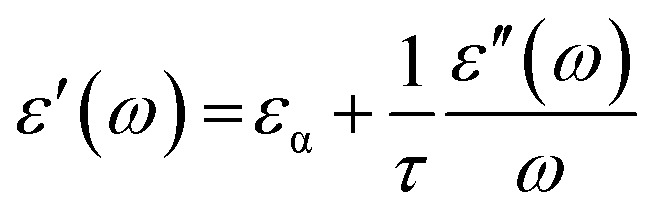
13
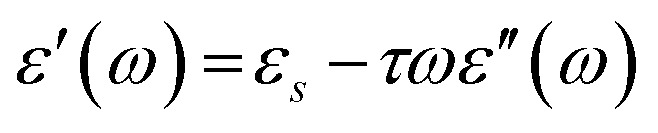
where, *ω* = 2π*ν* = angular frequency.

Straight lines with slope (=1/*τ*) and intercept (=*ε*_α_) , from eqn (12) and slope (=−*τ*) and intercept (=*ε*_s_), from eqn (13) were obtained which are similar to that obtained from fitting the dielectric spectra using non-linear least squares procedures based on the Levenberg–Marquardt algorithm, as seen in the Tables S7–S14 (ESI†).

## Conflicts of interest

There are no conflicts to declare.

## Supplementary Material

RA-010-C9RA08392H-s001
