# Supplementary material for: A dielectric and spectrophotometric study of the tautomerization of 2-hydroxypyridine and 2-mercaptopyridine in water
Source: RSC Adv. 2020 Jan 13;10(4):2389–95. doi: 10.1039/c9ra08392h (PMC9048638; doi:10.1039/c9ra08392h)
Supplement: RA-010-C9RA08392H-s001 [file RA-010-C9RA08392H-s001.pdf]

Electronic Supplementary Material (ESI) for RSC Advances.
This journal is © The Royal Society of Chemistry 2019

Supplementary Information:

A Dielectric and Spectrophotometric Study of the tautomerization of 2- Hydroxypyridine and 2-Mercaptopyridine in water

Biswadeep Bomzon, *Yashita, Khunger, Ranga Subramanian**

Department of Chemistry,

Indian Institute of Technology Patna, Bihar- 801106

India

Supporting Information Available

1) Sample preparation:

A. 15.9 mg of 2-hydroxypyridine was weighed using Mettler Toledo, ME204, and dissolved in triple-distilled water in a calibrated 10 ml volumetric flask to make a stock solution of 16.824 $X10^{-3}$ M. This stock solution was then diluted further to make 19 solutions of different concentrations from the range of 0.168 X 10^{-5} M to 11.777 X 10^{-5} M.

18.7 mg of 2- mercaptopyridine were weighed using the ME204, and 10 ml of 16.824 $X10^{-3}$M solutions were made, which was used as a stock solution to prepare different solutions from 0.168 X 10^{-5} M to 11.777 X 10^{-5} M, 2MPy solutions.

1.7µl 2- methoxypyridine was used to prepare the 10ml of 16.824 $X10^{-2}$M stock solution, which was then used to prepare a solution of 1.682 $X10^{-5}$M concentration for spectrophotometric determination of $pk_{1(OMe)}$.

17.7µl of 2- (methylthio) pyridine was used to prepare 10 ml of 16.824 $X10^{-2}$M stock solution, which was diluted to prepare 10 ml of 1.682 $X10^{-4}$M solutions for spectrophotometric determination of $pk_{1(SMe).}$ Similarly, 16.9µl of N- methyl - 2- pyridone was dissolved in 10 ml of triple distilled water to prepare 16.824 $X10^{-2}$M stock solution, and it was diluted to prepare 1.682 $X10^{-4}$M solutions for the determination of $pk_{1(NMe)}$.

2) Standardization of NaOH and HCl solutions:

Oxalic acid dihydrate (Sigma Aldrich, with ≥ 99%) was used to standardize the NaOH solutions using the phenolphthalein indicator. The same NaOH was then used to standardize the HCl solutions using the same indicator.

Table S1. Determination of the basic ionization constant or pk_1 of 2 – hydroxypyridine:

Concentration: 1.682 X 10^{-5} M.

Analytical wavelength = 270 nm. Cells: 1 cm.

pH for absorption of neutral molecule (A_M) = 10.12

pH for absorption of cation (A_I) = -1.68 [1]

$$pk_1 = pH + log\frac{A - A_M}{A_I - A}$$

Sl. No.	pH of Buffer	A_M	A_I	A	pk_1
1	0.75	0.0377	0.1145	0.0765	0.76
2	0.57	0.0377	0.1145	0.0854	0.78
3	0.97	0.0377	0.1145	0.0668	0.75

Average pk_1 = 0.76 ± 0.01, literature value = 0.75 [2]

Table S2. Determination of the acidic ionization constant or pk_2 of 2 – hydroxypyridine:

Concentration: 1.682 X 10^{-5} M.

Analytical wavelength = 234 nm. Cells: 1 cm.

pH for absorption of neutral molecule (A_M) = 2.72

pH for absorption of anion (A_I) = 13.84

$$pk_2 = pH + log\frac{A_I - A}{A - A_M}$$

Sl. No.	pH of Buffer	A_M	A_I	A	pk_2
1	11.59	0.0346	0.1251	0.0537	12.16
2	11.43	0.0346	0.1251	0.0498	12.12
3	11.83	0.0346	0.1251	0.0642	12.14

Average pk_2 = 12.14 ± 0.02, literature value = 11.62 [2]

Table S3. Determination of the acidic ionization constant or pk_2 of 2 – mercaptopyridine:

Concentration: 1.682 X 10^{-5} M.

Analytical wavelength = 255 nm. Cells: 1 cm.

pH for absorption of neutral molecule (A_M) = 2.86

pH for absorption of anion (A_I) = 12.58

$$pk_2 = pH + log\frac{A_I - A}{A - A_M}$$

Sl. No.	pH of Buffer	A_M	A_I	A	pk_2
1	9.80	0.0741	0.1783	0.1085	10.11
2	9.61	0.0741	0.1783	0.0998	10.09
3	10.00	0.0741	0.1783	0.1199	10.10

Average pk_2 = 10.10 ± 0.01, literature value = 9.81 [3]

Table S4. Determination of the basic ionization constant or $pk_{1(XMe)}$ of 2 – Methoxypyridine: (X = O)

Concentration: 1.682 X 10^{-5} M.

Analytical wavelength = 283 nm. Cells: 1 cm.

pH for absorption of neutral molecule (A_M) = 11.64

pH for absorption of anion (A_I) = 0.52

$$pk_{XMe} = pH + log\frac{A - A_M}{A_I - A}$$

Sl. No.	pH of Buffer	A_M	A_I	A	$pk_{1(XMe)}$
1	3.27	0.0023	0.0153	0.0087	3.26
2	3.08	0.0023	0.0153	0.010	3.24
3	3.49	0.0023	0.0153	0.0070	3.24

Average $pk_{1(XMe)}$ = 3.25 ± 0.01, literature value = 3.28 [2]

Table S5. Determination of the basic ionization constant or $pk_{1(NMe)}$ of N- Methyl- 2-Pyridone:

Concentration: 1.682 X 10^{-4} M.

Analytical wavelength = 272 nm. Cells: 1 cm.

pH for absorption of neutral molecule (A_M) = 11.54

pH for absorption of anion (A_I) = -1.68 [1]

$$pk_{NMe} = pH + log\frac{A - A_M}{A_I - A}$$

Sl. No.	pH of Buffer	A_M	A_I	A	$pk_{1(NMe)}$
1	0.33	0.0411	0.1233	0.0812	0.31
2	0.12	0.0411	0.1233	0.0917	0.32
3	0.52	0.0411	0.1233	0.0726	0.31

Average $pk_{1(NMe)}$ = 0.31 ± 0.01, literature value = 0.32 [2]

Table S6. Determination of the basic ionization constant or $pk_{1(XMe)}$ of 2- Methylthiopyridine: (X= S)

Concentration: 1.682 X 10^{-4} M.

Analytical wavelength = 283 nm. Cells: 1 cm.

pH for absorption of neutral molecule (A_M) = 11.60

pH for absorption of anion (A_I) = 1.07

$$pk_{XMe} = pH + log\frac{A_M - A}{A - A_I}$$

Sl. No.	pH of Buffer	A_M	A_I	A	$pk_{1(XMe)}$
1	3.59	0.0577	0.0226	0.0446	3.36
2	3.39	0.0577	0.0226	0.0405	3.37
3	3.78	0.0577	0.0226	0.0477	3.38

Average $pk_{1(XMe)}$ = 3.37 ± 0.01, literature value = 3.59 [4]

Table S7. Comparison of ε_α values obtained from the fitting of dielectric spectra to Debye equation (equation (5)) and equation (6) of 2- hydroxypyridine.

Sl. No.	Concentration (10^{-5} M)	Obtained from equation (5)	Obtained from equation (6)
1	Water	5.38(2) ± 0.10	5.6(4) ± 0.4
2	0.168	5.80(2) ± 0.77	8.7(2) ± 0.3
3	0.252	5.29(1) ± 0.30	7.0(1) ± 0.9
4	0.330	3.09(2) ± 3.02	6.9(2) ± 1.5
5	0.421	4.95(1) ± 0.21	6.3(2) ± 0.4
6	0.505	4.79(2) ± 0.18	6.7(1) ± 1.1
7	0.673	5.47(2) ± 0.50	6.3(2) ± 1.0
8	0.841	4.95(1) ± 0.37	7.1 (1) ± 1.3
9	1.001	5.50(1) ± 0.53	7.4(1) ± 0.9
10	1.178	5.06(2) ± 0.09	6.5(2) ± 0.7
11	1.346	5.18(1) ± 0.13	6.6(2) ± 0.9
12	1.430	4.72(1) ± 0.53	6.2(1) ± 0.5
13	1.514	4.49(1) ± 0.47	5.6(1) ± 0.9
14	1.682	5.11(1) ± 0.08	7.9(2) ± 0.2
15	3.365	4.92(1) ± 0.22	6.3(1) ± 0.4
16	5.047	5.13(1) ± 0.40	6.9(1) ± 0.7
17	6.730	4.94(1) ± 0.31	6.7(1) ± 0.3
18	8.412	5.06(1) ± 0.29	5.9(1) ± 1.2
19	10.090	5.37(1) ± 0.85	7.3(2) ± 0.9
20	11.777	5.08(2) ± 0.07	8.2(2) ± 1.0
21	50.000	5.50 (2) ± 0.39	6.4 (2) ± 0.4
22	100.00	5.80 (2) ± 0.54	7.3 (3) ± 0.4
23	500.00	5.89 (2) ± 0.56	6.8 (2)± 0.4
24	1000.0	5.76 (1) ± 0.23	6.7 (1)± 0.4

Table S8. Comparison of $\Delta\varepsilon$ values obtained from the fitting of dielectric spectra to equation (5) and taking a difference of ε_s (from Equation (7)) and ε_α (from Equation (6)) of 2-hydroxypyridine.

Sl. No.	Concentration (10^{-5} M)	Obtained from equation (5)	Obtained from $\Delta\varepsilon = \varepsilon_s - \varepsilon_\alpha$
1	Water	73.24(2) ± 0.07	73.0(4) ± 0.3
2	0.168	73.63(1) ± 0.58	70.8(2) ± 0.7
3	0.252	73.50(1) ± 0.31	71.8(1) ± 1.1
4	0.330	73.97(2) ± 0.36	70.1(2) ± 2.2
5	0.421	73.90(1) ± 0.72	72.5(2) ± 0.3
6	0.505	72.55(1) ± 0.39	71.6(1) ± 1.0
7	0.673	72.83(2) ± 0.20	71.8(2) ± 0.5
8	0.841	73.03(1) ± 0.49	70.9(1) ± 0.5
9	1.001	73.06(1) ± 0.61	71.2(1) ± 0.6
10	1.178	73.55(2) ± 0.30	72.1(2) ± 0.4
11	1.346	73.44(1) ± 0.41	72.0(2) ± 0.9
12	1.430	73.60(1) ± 0.44	73.1(1) ± 0.5
13	1.514	73.47(1) ± 0.79	71.1(1) ± 1.9
14	1.682	72.92(1) ± 0.39	70.1(2) ± 0.6
15	3.365	73.20(1) ± 0.27	71.7(1) ± 0.5
16	5.047	73.48(1) ± 0.32	71.7(1) ± 0.9
17	6.730	73.41(1) ± 0.56	71.7(1) ± 0.2
18	8.412	73.21(1) ± 0.72	72.3(1) ± 0.7
19	10.090	73.04(1) ± 0.36	71.1(2) ± 0.6
20	11.777	73.38(1) ± 0.45	70.3(2) ± 1.3
21	50.000	73.23(2)±0.57	72.3(3) ± 0.5
22	100.00	73.64(2)±0.44	71.1(2) ± 0.3
23	500.00	72.75(2)±0.61	71.8(1) ± 0.4
24	1000.0	72.63(1)±0.59	71.7(3) ± 0.3

Table S9. Comparison of ε_s values obtained from the fitting of dielectric spectra to equation (5) and equation (7) of 2- hydroxypyridine.

Sl. No.	Concentration (10^{-5} M)	Obtained from equation (5)	Obtained from equation (7)
1	Water	78.61(4) ± 0.17	78.64(1) ± 0.18
2	0.168	79.43(3) ± 0.70	79.42(1) ± 0.68
3	0.252	78.80(2) ± 0.58	78.81(4) ± 0.57
4	0.330	77.05(4) ± 2.69	77.05(1) ± 2.70
5	0.421	78.86(2) ± 0.59	78.85(2) ± 0.59
6	0.505	78.34(3) ± 0.48	78.34(1) ± 0.48
7	0.673	78.22(4) ± 0.42	78.13(1) ± 0.51
8	0.841	77.99(2) ± 0.86	77.99(5) ± 0.84
9	1.001	78.57(2) ± 0.62	78.56(1) ± 0.62
10	1.178	78.62(4) ± 0.38	78.63(1) ± 0.37
11	1.346	78.62(2) ± 0.54	78.64(2) ± 0.53
12	1.430	78.31(2) ± 0.14	78.36(4) ± 0.11
13	1.514	77.96(2) ± 1.20	78.97(2) ± 0.39
14	1.682	78.02(2) ± 0.46	78.04(4) ± 0.45
15	3.365	78.12(2) ± 0.43	77.93(3) ± 0.13
16	5.047	78.61(2) ± 0.39	78.61(4) ± 0.38
17	6.730	78.34(2) ± 0.26	78.36(2) ± 0.26
18	8.412	78.28(2) ± 0.54	78.27(3) ± 0.52
19	10.090	78.42(2) ± 0.50	78.39(4) ± 0.49
20	11.777	78.46(3) ± 0.39	78.47(1) ± 0.39
21	50.000	78.74(2)±0.08	78.70(3) ±0.43
22	100.00	78.45(2)±0.24	78.24(2)±0.16
23	500.00	78.65(2)±0.23	78.59(1)±0.04
24	1000.0	78.38(1)±0.39	78.46(3)±0.05

Table S10. Comparison of τ values obtained from the fitting of dielectric spectra to equation (5) and equation (6) and (7) of solutions 2- hydroxypyridine.

Sl. No.	Concentration (10^{-5} M)	Obtained from equation (5)	Obtained from equation (6)	Obtained from equation (7)
1	Water	8.320(3) ± 0.015	8.356(1) ± 0.068	8.321(3) ± 0.018
2	0.168	8.273(3) ± 0.129	8.527(2) ± 0.171	8.267(2) ± 0.115
3	0.252	7.747(1) ± 0.227	8.166(5) ± 0.151	7.962(1) ± 0.262
4	0.330	7.702(3) ± 0.392	8.272(2) ± 0.405	7.686(3) ± 0.397
5	0.421	7.973(2) ± 0.083	8.072(4) ± 0.098	7.979(2) ± 0.083
6	0.505	8.013(3) ± 0.189	8.298(3) ± 0.362	8.022(2) ± 0.181
7	0.673	8.017(3) ± 0.199	8.129(3) ± 0.286	8.011(3) ± 0.173
8	0.841	8.053(2) ± 0.115	8.385(3) ± 0.270	8.056(2) ± 0.111
9	1.001	7.997(2) ± 0.188	8.268(5) ± 0.223	7.995(2) ± 0.190
10	1.178	7.927(3) ± 0.109	8.141(2) ± 0.225	7.952(3) ± 0.163
11	1.346	7.977(1) ± 0.121	8.187(3) ± 0.012	7.986(1) ± 0.115
12	1.430	8.047(2) ± 0.115	8.242(3) ± 0.150	8.075(2) ± 0.114
13	1.514	8.017(2) ± 0.140	8.178(4) ± 0.206	8.028(2) ± 0.130
14	1.682	8.007(2) ± 0.060	8.433(2) ± 0.093	8.019(2) ± 0.057
15	3.365	8.040(1) ± 0.127	8.240(3) ± 0.067	8.047(1) ± 0.115
16	5.047	8.017(1) ± 0.142	8.304(5) ± 0.132	8.023(1) ± 0.131
17	6.730	7.877(1) ± 0.165	8.120(3) ± 0.134	7.889(1) ± 0.156
18	8.412	8.067(1) ± 0.146	8.190(1) ± 0.297	8.064(1) ± 0.293
19	10.09	7.977(2) ± 0.311	8.253(3) ± 0.288	7.985(2) ± 0.015
20	11.777	8.093(3) ± 0.144	8.586(2) ± 0.054	8.095(2) ± 0.015
21	50.000	8.340(2) ± 0.062	8.479(3) ± 0.059	8.333(4) ± 0.066
22	100.00	8.407(1) ± 0.020	8.649(4) ± 0.114	8.398(1) ± 0.028
23	500.00	8.390(3) ± 0.071	8.529(1) ± 0.092	8.380(2) ± 0.069
24	1000.0	8.343(3) ± 0.104	8.479(2) ± 0.120	8.339(1) ± 0.102

Table S11. Comparison of ε_α values obtained from the fitting of dielectric spectra to equation (5) and equation (6) of 2- mercaptopyridine.

Sl. No.	Concentration (10^{-5} M)	Obtained from equation (5)	Obtained from equation (6)
1	Water	5.38(2) ± 0.10	5.6(4) ± 0.4
2	0.168	4.80(2) ± 0.35	6.0(1) ± 0.8
3	0.252	5.52(4) ± 0.31	6.3(2) ± 0.5
4	0.336	5.49(3) ± 1.20	6.3(1) ± 2.7
5	0.421	5.32(3) ± 0.16	7.0(2) ± 0.2
6	0.505	5.75(2) ± 0.31	6.3(1) ± 0.4
7	0.673	4.72(3) ± 0.27	8.5(3) ± 0.7
8	0.841	5.07(3) ± 0.42	6.3(1) ± 1.0
9	1.009	4.79(3) ± 0.88	5.9(1) ± 1.7
10	1.178	5.69(2) ± 0.29	5.9(4) ± 0.7
11	1.346	5.43(3) ± 0.49	8.0(2) ± 0.9
12	1.430	4.86(2) ± 0.01	5.7(1) ± 0.1
13	1.514	5.52(3) ± 0.14	7.4(2) ± 0.2
14	1.682	4.73(3) ± 0.07	5.9(2) ± 0.0
15	3.365	5.03(2) ± 0.12	5.6(1) ± 0.2
16	5.047	4.76(1) ± 0.03	5.7(5) ± 1.8
17	6.730	5.04(2) ± 0.09	5.2(1) ± 0.2
18	8.412	5.80(4) ± 0.42	6.7(1) ± 1.3
19	10.094	5.56(3) ± 0.20	7.7(2) ± 0.2
20	11.776	4.80(3) ± 0.30	4.6(1) ± 0.8
21	50.000	5.24(2) ±0.57	6.3(3) ± 0.9
22	100.00	4.65(2) ±0.44	6.1(2) ± 1.1
23	500.00	4.87(2) ±0.61	6.6(1) ± 0.9
24	1000.0	5.43(1) ±0.59	6.1(3) ± 0.5

Table S12. Comparison of $\Delta\varepsilon$ values obtained from the fitting of dielectric spectra to equation (5) and taking a difference of ε_s (from Equation (7)) and ε_α (from Equation (6)) of 2-mercaptopyridine.

Sl. No.	Concentration (10^{-5} M)	Obtained from equation (5)	Obtained from $\Delta\varepsilon = \varepsilon_s - \varepsilon_\alpha$
1	Water	73.24(2) ± 0.07	73.0(4) ± 0.3
2	0.168	73.15(1) ± 0.40	71.9(1) ± 0.7
3	0.252	74.06(3) ± 0.80	73.3(2) ± 1.0
4	0.336	73.37(3) ± 1.70	72.5(1) ± 3.3
5	0.421	73.40(2) ± 0.17	71.7(4) ± 0.2
6	0.505	72.61(1) ± 0.47	72.0(2) ± 0.5
7	0.673	73.34(2) ± 0.48	69.6(3) ± 1.0
8	0.841	73.13(3) ± 0.90	71.8(1) ± 1.5
9	1.009	73.47(2) ± 1.32	72.2(1) ± 2.2
10	1.178	73.24(1) ± 0.41	72.9(5) ± 0.8
11	1.346	72.04(3) ± 0.52	69.3(2) ± 0.9
12	1.430	73.55(2) ± 0.20	72.6(1) ± 0.2
13	1.514	72.71(3) ± 0.22	70.7(2) ± 0.2
14	1.682	74.38(2) ± 0.26	73.4(2) ± 0.4
15	3.365	73.56(2) ± 0.31	73.0(1) ± 0.2
16	5.047	73.20(1) ± 1.05	72.3(1) ± 2.9
17	6.730	73.69(2) ± 0.17	73.6(1) ± 0.5
18	8.412	72.20(3) ± 1.30	71.3(1) ± 2.3
19	10.094	72.14(2) ± 0.34	69.9(2) ± 0.4
20	11.776	74.07(2) ± 0.60	74.3(1) ± 1.2
21	50.000	73.48(2) ±0.57	72.4(3) ± 0.8
22	100.00	73.54(2) ±0.44	72.2(2) ± 1.0
23	500.00	72.73(2) ±0.61	72.0(1) ± 0.9
24	1000.0	73.03(1) ±0.59	72.4(3) ± 0.6

Table S13. Comparison of ε_s values obtained from the fitting of dielectric spectra to equation (5) and equation (7) of 2- mercaptopyridine.

Sl. No.	Concentration (10^{-5} M)	Obtained from equation (5)	Obtained from equation (7)
1	Water	78.61(4) ± 0.17	78.64(1) ± 0.18
2	0.168	77.95(3) ± 0.16	77.94(1) ± 0.09
3	0.252	79.57(1) ± 0.52	79.59(2) ± 0.24
4	0.336	78.83(1) ± 0.48	78.82(1) ± 0.34
5	0.421	78.73(5) ± 0.05	78.73(1) ± 0.03
6	0.505	78.37(3) ± 0.42	78.32(1) ± 0.17
7	0.673	78.07(5) ± 0.24	78.06(1) ± 0.06
8	0.841	78.20(1) ± 0.51	78.17(1) ± 0.26
9	1.009	78.23(5) ± 0.52	78.22(1) ± 0.26
10	1.178	78.93(3) ± 0.24	78.59(1) ± 0.05
11	1.346	77.61(1) ± 0.15	77.38(1) ± 0.4
12	1.430	78.41(3) ± 0.19	78.39(1) ± 0.04
13	1.514	78.22(1) ± 0.14	78.21(1) ± 0.02
14	1.682	79.12(5) ± 0.30	79.11(1) ± 0.09
15	3.365	78.90(4) ± 0.07	78.61(1) ± 0.19
16	5.047	77.87(3) ± 1.13	77.95(1) ± 1.09
17	6.730	78.74(4) ± 0.26	78.74(1) ± 0.07
18	8.412	78.00(1) ± 0.99	78.00(1) ± 1.01
19	10.094	77.71(5) ± 0.18	77.66(2) ± 0.04
20	11.776	78.87(5) ± 0.43	78.87(1) ± 0.20
21	50.000	78.72(2)±0.44	78.70(3) ± 0.4
22	100.00	78.18(2)±0.10	78.24(2) ± 0.2
23	500.00	78.60(2)±0.04	78.59(1) ± 0.1
24	1000.0	78.46(1)±0.21	78.46(3) ± 0.2

Table S14. Comparison of τ values obtained from the fitting of dielectric spectra to equation (5) and equation (6) and (7) of solutions 2- mercaptopyridine.

Sl. No.	Concentration (10^{-5} M)	Obtained from equation (5)	Obtained from equation (6)	Obtained from equation (7)
1	Water	8.320(3) ± 0.015	8.356(1) ± 0.068	8.321(3) ± 0.018
2	0.168	8.043(2) ± 0.115	8.242(4) ± 0.058	8.041(2) ± 0.106
3	0.252	8.337(1) ± 0.104	8.432(2) ± 0.119	8.350(1) ± 0.104
4	0.330	8.150(5) ± 0.265	8.311(3) ± 0.141	8.127(5) ± 0.208
5	0.421	8.360(5) ± 0.037	8.633(2) ± 0.534	8.383(4) ± 0.063
6	0.505	8.280(3) ± 0.043	8.373(4) ± 0.017	8.253(3) ± 0.035
7	0.673	8.030(4) ± 0.082	8.631(2) ± 0.064	8.025(4) ± 0.078
8	0.841	8.093(5) ± 0.074	8.300(4) ± 0.170	8.079(1) ± 0.062
9	1.001	8.040(4) ± 0.115	8.238(1) ± 0.185	8.019(5) ± 0.108
10	1.178	8.283(3) ± 0.083	8.323(1) ± 0.268	8.268(3) ± 0.069
11	1.346	8.047(5) ± 0.085	8.488(2) ± 0.140	7.994(5) ± 0.066
12	1.430	8.053(3) ± 0.009	8.188(4) ± 0.172	8.045(3) ± 0.004
13	1.514	8.293(5) ± 0.062	8.608(2) ± 0.011	8.280(5) ± 0.058
14	1.682	8.230(4) ± 0.008	8.400(5) ± 0.078	8.231(4) ± 0.008
15	3.365	8.137(3) ± 0.020	8.225(4) ± 0.001	8.138(4) ± 0.024
16	5.047	8.137(2) ± 0.009	8.328(1) ± 0.039	8.167(2) ± 0.007
17	6.730	8.197(3) ± 0.012	8.205(3) ± 0.328	8.199(3) ± 0.016
18	8.412	8.347(1) ± 0.084	8.501(4) ± 0.038	8.346(5) ± 0.068
19	10.090	8.120(4) ± 0.064	8.493(3) ± 0.214	8.094(4) ± 0.060
20	11.777	8.133(4) ± 0.031	8.095(4) ± 0.068	8.147(4) ± 0.032
21	50.000	8.353(2)±0.034	8.527(3)± 0.149	8.344(3) ± 0.037
22	100.00	8.293(2)±0.063	8.453(2)± 0.188	8.285(3) ± 0.048
23	500.00	8.296(2)±0.061	8.579(1)± 0.132	8.291(3) ± 0.063
24	1000.0	8.333(1)±0.101	8.465(3)± 0.032	8.364(3) ± 0.104

Table S15. High-frequency dielectric permittivity, ε_α, relaxation amplitude $\Delta\varepsilon$, static permittivity, ε_s, relaxation time, τ of Debye model fitted to the dielectric spectra of 2HPy at room temperature. Reduced error function, χ^2 in all the solutions were close to unity.

Sl. No	Conc.(10^{-5} M)	ε_α	$\Delta\varepsilon$	ε_s	τ
1	Water	5.38(2) ± 0.10	73.24(2) ± 0.07	78.61(4) ± 0.17	8.320(3) ± 0.015
2	0.168	5.80(2) ± 0.77	73.63(1) ± 0.58	79.43(3) ± 0.70	8.273(3) ± 0.129
3	0.252	5.29(1) ± 0.30	73.50(1) ± 0.31	78.80(2) ± 0.58	7.747(1) ± 0.227
4	0.330	3.09(2) ± 3.02	73.97(2) ± 0.36	77.05(4) ± 2.69	7.702(3) ± 0.392
5	0.421	4.95(1) ± 0.21	73.90(1) ± 0.72	78.86(2) ± 0.59	7.973(2) ± 0.083
6	0.505	4.79(2) ± 0.18	72.55(1) ± 0.39	78.34(3) ± 0.48	8.013(3) ± 0.189
7	0.673	5.47(2) ± 0.50	72.83(2) ± 0.20	78.22(4) ± 0.42	8.017(3) ± 0.199
8	0.841	4.95(1) ± 0.37	73.03(1) ± 0.49	77.99(2) ± 0.86	8.053(2) ± 0.115
9	1.001	5.50(1) ± 0.53	73.06(1) ± 0.61	78.57(2) ± 0.62	7.997(2) ± 0.188
10	1.178	5.06(2) ± 0.09	73.55(2) ± 0.30	78.62(4) ± 0.38	7.927(3) ± 0.109
11	1.346	5.18(1) ± 0.13	73.44(1) ± 0.41	78.62(2) ± 0.54	7.977(1) ± 0.121
12	1.430	4.72(1) ± 0.53	73.60(1) ± 0.44	78.31(2) ± 0.14	8.047(2) ± 0.115
13	1.514	4.49(1) ± 0.47	73.47(1) ± 0.79	77.96(2) ± 1.20	8.017(2) ± 0.140
14	1.682	5.11(1) ± 0.08	72.92(1) ± 0.39	78.02(2) ± 0.46	8.007(2) ± 0.060
15	3.365	4.92(1) ± 0.22	73.20(1) ± 0.27	78.12(2) ± 0.43	8.040(1) ± 0.127
16	5.047	5.13(1) ± 0.40	73.48(1) ± 0.32	78.61(2) ± 0.39	8.017(1) ± 0.142
17	6.730	4.94(1) ± 0.31	73.41(1) ± 0.56	78.34(2) ± 0.26	7.877(1) ± 0.165
18	8.412	5.06(1) ± 0.29	73.21(1) ± 0.72	78.28(2) ± 0.54	8.067(1) ± 0.146
19	10.090	5.37(1) ± 0.85	73.04(1) ± 0.36	78.42(2) ± 0.50	7.977(2) ± 0.311
20	11.777	5.08(2) ± 0.07	73.38(1) ± 0.45	78.46(3) ± 0.39	8.093(3) ± 0.144
21	50.000	5.50(2) ± 0.39	73.23(2)±0.57	78.74(2)±0.08	8.340(2) ± 0.062
22	100.00	5.80(2) ± 0.54	73.64(2)±0.44	78.45(2)±0.24	8.407(1) ± 0.020
23	500.00	5.89(2) ± 0.56	72.75(2)±0.61	78.65(2)±0.23	8.390(3) ± 0.071
24	1000.0	5.76(1) ± 0.23	72.63(1)±0.59	78.38(1)±0.39	8.343(3) ± 0.104

Table S16. High-frequency dielectric permittivity, ε_α, relaxation amplitude $\Delta\varepsilon$, static permittivity, ε_s, relaxation time, τ of Debye model fitted to the dielectric spectra of 2MPy at room temperature. Reduced error function, χ^2 in all the solutions were close to unity

Sl. No	Conc. (10^{-5} M)	ε_α	$\Delta\varepsilon$	ε_s	τ
1	Water	5.38(2) ± 0.10	73.24(2) ± 0.07	78.61(4) ± 0.17	8.320(3) ± 0.015
2	0.168	4.80(2) ± 0.35	73.15(1) ± 0.40	77.95(3) ± 0.16	8.043(2) ± 0.115
3	0.252	5.52(4) ± 0.31	74.06(3) ± 0.80	79.57(1) ± 0.52	8.337(1) ± 0.104
4	0.336	5.49(3) ± 1.20	73.37(3) ± 1.70	78.83(1) ± 0.48	8.150(5) ± 0.265
5	0.421	5.32(3) ± 0.16	73.40(2) ± 0.17	78.73(5) ± 0.05	8.360(5) ± 0.037
6	0.505	5.75(2) ± 0.31	72.61(1) ± 0.47	78.37(3) ± 0.42	8.280(3) ± 0.043
7	0.673	4.72(3) ± 0.27	73.34(2) ± 0.48	78.07(5) ± 0.24	8.030(4) ± 0.082
8	0.841	5.07(3) ± 0.42	73.13(3) ± 0.90	78.20(1) ± 0.51	8.093(5) ± 0.074
9	1.009	4.79(3) ± 0.88	73.47(2) ± 1.32	78.23(5) ± 0.52	8.040(4) ± 0.115
10	1.178	5.69(2) ± 0.29	73.24(1) ± 0.41	78.93(3) ± 0.24	8.283(3) ± 0.083
11	1.346	5.43(3) ± 0.49	72.04(3) ± 0.52	77.61(1) ± 0.15	8.047(5) ± 0.085
12	1.430	4.86(2) ± 0.01	73.55(2) ± 0.20	78.41(3) ± 0.19	8.053(3) ± 0.009
13	1.514	5.52(3) ± 0.14	72.71(3) ± 0.22	78.22(1) ± 0.14	8.293(5) ± 0.062
14	1.682	4.73(3) ± 0.07	74.38(2) ± 0.26	79.12(5) ± 0.30	8.230(4) ± 0.008
15	3.365	5.03(2) ± 0.12	73.56(2) ± 0.31	78.90(4) ± 0.07	8.137(3) ± 0.020
16	5.047	4.76(1) ± 0.03	73.20(1) ± 1.05	77.87(3) ± 1.13	8.137(2) ± 0.009
17	6.730	5.04(2) ± 0.09	73.69(2) ± 0.17	78.74(4) ± 0.26	8.197(3) ± 0.012
18	8.412	5.80(4) ± 0.42	72.20(3) ± 1.30	78.00(1) ± 0.99	8.347(1) ± 0.084
19	10.094	5.56(3) ± 0.20	72.14(2) ± 0.34	77.71(5) ± 0.18	8.120(4) ± 0.064
20	11.776	4.80(3) ± 0.30	74.07(2) ± 0.60	78.87(5) ± 0.43	8.133(4) ± 0.031
21	50.000	5.24(2) ±0.57	73.48(2)±0.57	78.72(2)±0.44	8.353(2)±0.034
22	100.00	4.65(2) ±0.44	73.54(2)±0.44	78.18(2)±0.10	8.293(2)±0.063
23	500.00	4.87(2) ±0.61	72.73(2)±0.61	78.60(2)±0.04	8.296(2)±0.061
24	1000.0	5.43(1) ±0.59	73.03(1)±0.59	78.46(1)±0.21	8.333(1)±0.101

References:

1. A. Albert and E. P. Serjeant, *The determination of ionization constants: a laboratory manual*, Chapman and Hall, London, 1984.

2. A. Albert and J. N. Phillips, *J. Chem.Soc.,(Resumed)*, 1956, DOI: 10.1039/JR9560001294, 1294-1304

3. R. A. Jones and A. R. Katritzky, *J. Chem. Soc. (Resumed)*, 1958, **0**, 3610-3613.

4. A. Habibi-Yangjeh, E. Pourbasheer, and M. Danandeh-Jenagharad, *Monatsh.Chem.,* 2009, **140**, 15-27.
